# Duplication of clostridial binding domains for enhanced macromolecular delivery into neurons

**DOI:** 10.1016/j.toxcx.2019.100019

**Published:** 2019-12-28

**Authors:** Charlotte Leese, Rebecca Bresnahan, Ciara Doran, Deniz Simsek, Alexander D. Fellows, Laura Restani, Matteo Caleo, Giampietro Schiavo, Timur Mavlyutov, Tina Henke, Thomas Binz, Bazbek Davletov

**Affiliations:** a Department of Biomedical Science, University of Sheffield, Sheffield, S10 2TN, UK; b Department of Neuromuscular Diseases, UCL Queen Square Institute of Neurology, Queen Square, London, WC1N 3BG, UK; c CNR Neuroscience Institute, Pisa, 1-56124 Pisa, Italy; d UK Dementia Research Institute, University College London, London, WC1E 6BT, UK; e Department of Ophthalmology and Visual Sciences, University of Wisconsin-Madison, Madison, WI, 53706, USA; f Institute of Cellular Biochemistry, Hannover Medical School, Hannover, 30625, Germany

**Keywords:** Botulinum, Tetanus, Duplicated, Double, Multivalent, Neuronal delivery

## Abstract

Neurological diseases constitute a quarter of global disease burden and are expected to rise worldwide with the ageing of human populations. There is an increasing need to develop new molecular systems which can deliver drugs specifically into neurons, non-dividing cells meant to last a human lifetime. Neuronal drug delivery must rely on agents which can recognise neurons with high specificity and affinity. Here we used a recently introduced ‘stapling’ system to prepare macromolecules carrying duplicated binding domains from the clostridial family of neurotoxins. We engineered individual parts of clostridial neurotoxins separately and combined them using a strong alpha-helical bundle. We show that combining two identical binding domains of tetanus and botulinum type D neurotoxins, in a sterically defined way by protein stapling, allows enhanced intracellular delivery of molecules into neurons. We also engineered a botulinum neurotoxin type C variant with a duplicated binding domain which increased enzymatic delivery compared to the native type C toxin. We conclude that duplication of the binding parts of tetanus or botulinum neurotoxins will allow production of high avidity agents which could deliver imaging reagents and large therapeutic enzymes into neurons with superior efficiency.

## Highlights

• Macromolecules carrying duplicated clostridial binding domains (Hc) were produced.
• Double tetanus Hc increased protein delivery into cultured rodent neurones.
• Double tetanus Hc increased enzyme delivery into rodent spinal cord and brain area.
• Double BoNT/D Hc increased enzyme delivery into rat and human neurones in culture.
• Recombinant double-Hc BoNT/C was engineered, increasing delivery in cell cultures.

## 1 Introduction

The intractable nature of many neurological disorders demands new approaches to deliver medications into neurons with high precision. Neurons are highly specialised cells carrying a unique set of lipids, carbohydrates and proteins, which together help to achieve the primary goal of neurons – fast transmission of information. The neuronal membrane is rich in complex gangliosides such as GD1a and GT1b which play an essential role in myelination of long, fast conducting axons (Vyas and Schnaar, 2001). Neurons are also rich in fast-conducting ion channels and proteins governing synaptic vesicle formation, loading of vesicles with neurotransmitters and stimulus-driven release of neurotransmitters from nerve endings. In the case of the neuromuscular junction, the release of acetylcholine results in immediate muscle contraction, whereas in CNS the release of glutamate, for example, leads to fast propagation of information. Prior to neurotransmitter release, synaptic vesicles fuse with the plasma membrane and a set of three proteins plays the critical role in this process of fusion (Sollner et al., 1993). The three proteins are syntaxin and SNAP-25 on the plasma membrane, and vesicle-associated membrane protein (VAMP), also known as synaptobrevin (Sollner et al., 1993). These three proteins, Soluble N-ethylmaleimide-sensitive factor Attachment Protein REceptors (SNAREs), form a tight helical bundle which bridges vesicular and plasma membranes, causing the two lipid bilayers to merge with subsequent release of neurotransmitters (Sutton et al., 1998). Identification of the SNARE proteins in the early 1990s led to a breakthrough in our understanding of the actions of the most potent toxins known to man – clostridial neurotoxins, tetanus toxin and botulinum neurotoxins (BoNTs), all of which cause long-lasting and yet reversible paralysis (Rossetto et al., 2014, Hayashi et al., 1994). The family of neuron-specific BoNTs consist of seven classical serotypes (A-G), each cleaving a specific SNARE protein to cause a long-lasting flaccid paralysis, which may result in death (Rossetto et al., 2014, Hayashi et al., 1994). BoNTs type A, C and E cleave SNAP-25, while BoNTs type B, D, F and G cleave VAMPs, vesicle-associated membrane proteins (Binz, 2013). Unlike BoNTs, tetanus toxin cleaves neuronal VAMP proteins in the spinal cord after hijacking the retrograde transport operating inside motor neurons and this cleavage of VAMPs lead to spastic paralysis (Surana et al., 2018).

Clostridial neurotoxins target only neurons without any apparent cross-binding to other cells. These neurotoxins are precisely engineered proteins composed of three domains with their specific autonomous functions (Montal, 2010). Both tetanus toxin and BoNTs are 150-kDa proteins consisting of a 50-kDa enzymatic SNARE-cleaving light chain (**LC**) linked by a disulphide bond to a 100-kDa heavy chain, which contains two major structural domains – binding and translocation domains (Montal, 2010). The binding domain, namely C-terminal half of the heavy chain (**Hc**), binds to neuronal receptors – gangliosides GT1b and GD1a, and synaptic vesicle proteins, the latter becoming exposed on neurons after release of transmitters and before recovery by endocytosis (Surana et al., 2018, Montal, 2010). Once in the endocytic vesicle, the N-terminal half of the heavy chain (**Hn**) forms a channel in the endosome membrane through which the LC escapes into the presynaptic cytosol, while the reducing cytosolic environment severs the disulphide bond (Montal, 2010). In the case of BoNT/A, LC diffuses throughout the neuronal cytosol and cleaves the plasma membrane-associated SNAP-25 resulting in nerve block (Schiavo et al., 1993, Binz et al., 1994). For simplicity, the botulinum neurotoxin structure can be described as LCHn/Hc where Hc represents the binding module.

Among the seven BoNTs, type A (BoNT/A) has proved successful in alleviating symptoms of over 100 medical conditions arising from nerve over-activity, e.g., dystonias, spasticity, chronic migraine and autonomic hypersecretory disorders (Jankovic, 2004). As mentioned above, BoNT-induced nerve blockade is a direct consequence of cleavage of SNARE proteins: in the case of BoNT/A, it deletes just 9 amino acids from the C terminus of SNAP-25 preventing the merger of vesicular and plasma membranes (Schiavo et al., 1993, Binz et al., 1994). The removal of 9 amino acids does not prevent the SNARE bundle formation thereby trapping vesicles in a docked state until the neuron regenerates the whole nerve terminal, involving membrane remodelling (Ovsepian et al., 2019). The lasting persistence of cleaved SNAP-25 in BoNT/A-intoxicated neurons allowed a design of a unique antibody which recognises the BoNT/A-generated cleaved end of SNAP-25 (Yadirgi et al., 2017). This neoepitope antibody proved to be useful to observe the location of BoNT/A action both *in vitro* and in vivo and we exploited this antibody to study the effects of delivering botulinum enzyme using duplicated clostridial binding domains.

Duplication of binding domains is often observed in nature where high avidity is required for protein function (Vauquelin and Charlton, 2013). Examples of multiplication of binding domains occurring in natural molecules include antibodies, lectins and protein toxins (Vauquelin and Charlton, 2013, Roy et al., 2016, Davletov et al., 2012). We now show the functional effects of artificially duplicating the binding domains of tetanus and botulinum type C and D domains as prototype vehicles for delivery of drugs into the CNS. Our results show that a mere duplication of the Hc domains causes highly significant escalation of neuronal delivery of small molecules and enzymatic activity. We also observed an increase in the speed of action of novel botulinum constructs, which could be exploited for potential therapeutic benefits.

## 2 Results

### 2.1 Duplication of the tetanus binding domain

**Fig. 1:**
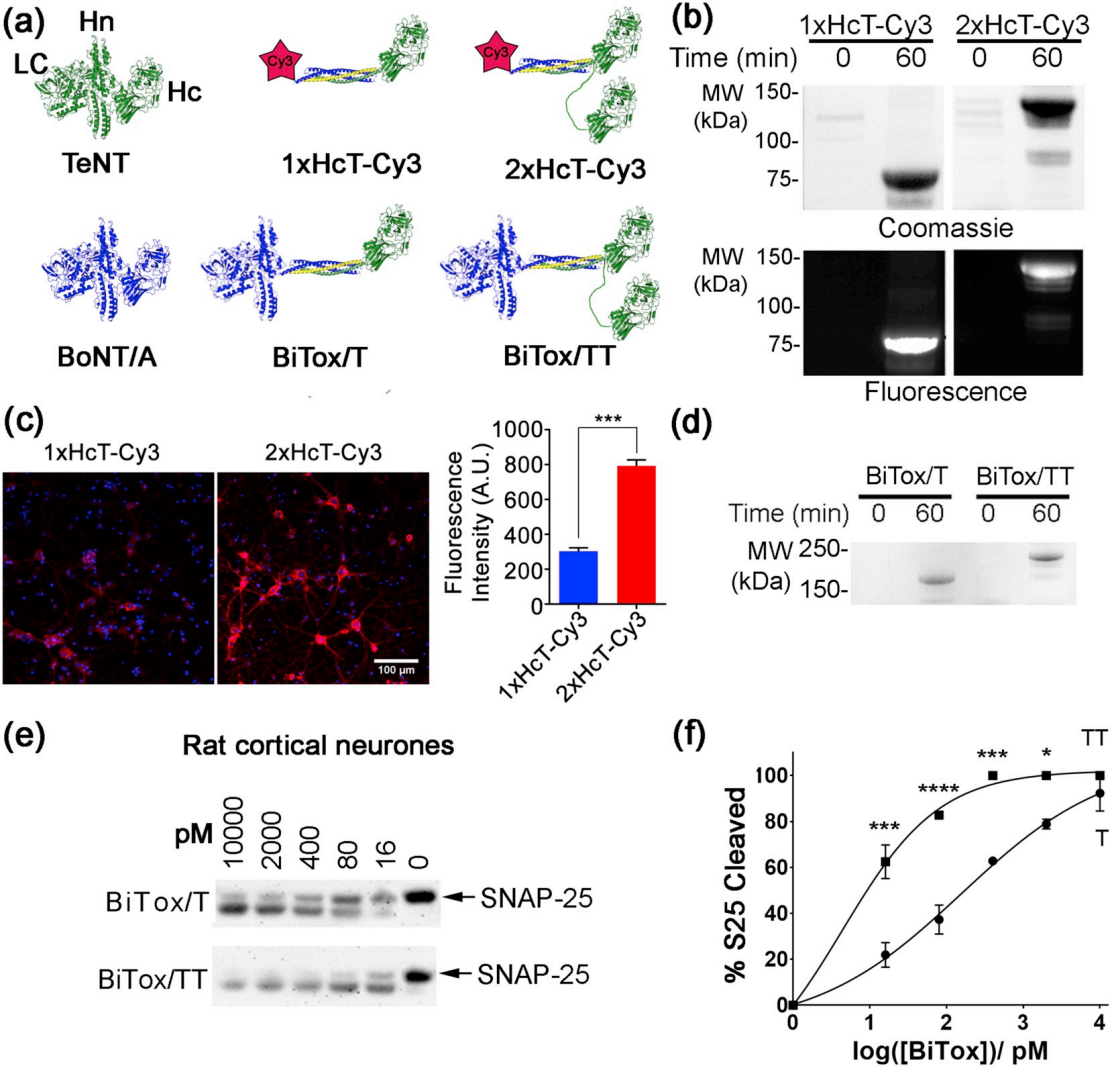
Duplication of tetanus binding domain results in increased binding to neurons and augmented cleavage of SNAP-25 in neurons. **a)** Schematic showing structural features of tetanus and botulinum neurotoxins together with chimeric proteins used. The coloured bridge is formed by three linking polypeptides**.** Red star indicates Cy3 fluorophore chemically attached to one of the linking peptides. **b)** SDS-PAGE gel showing 1xHcT-Cy3 and 2xHcT-Cy3 after the 60 min assembly reaction. Proteins were visualised by Coomassie staining (upper panel) and fluorescence (lower panel). **c)** Examples of fluorescent micrographs (left) and their quantification (right) of cultured rat cortical neurons treated with either 1xHcT-Cy3 or 2xHcT-Cy3 (both 10 nM, red). Nuclear staining was done using Hoechst 3342 stain (blue). Bar chart shows mean Cy3 intensity (red, *n* = 3). **d)** Coomassie-stained SDS-PAGE gel showing formation of Bitox/T and Bitox/TT and the difference in their apparent molecular weights. Proteins were analysed in non-reducing conditions and thus exhibit full protein content. **e)** Duplication of tetanus binding domain increases SNAP-25 cleaving activity of tetanus-botulinum type A stapled chimera. Example immunoblot of rat cortical neurons treated with either Bitox/T or Bitox/TT at the indicated concentrations (n = 3). **f)** Immunoanalysis reveals significant enhancement of the cleavage of SNAP-25 (S25) for Bitox/TT: BiTox/T = 120 pM, BiTox/TT = 4.4 pM (n = 3).

Our molecular stapling system utilises a truncated SNARE bundle and allows on-demand combination of protein parts upon simple mixing (Darios et al., 2010). For protein linking we used three shortened SNARE helical polypeptides which we call here linkers 1, 2 and 3 (green, yellow and blue). These three linkers assemble spontaneously within 1 h into a highly stable helical bundle permitting on-demand conjugation of proteins (Fig. 1).

The binding domain of tetanus neurotoxin (HcT) has been used by many groups for delivery of imaging reagents and therapeutics due to its well-known ability to bind motor neurons (Surana et al., 2018, Toivonen et al., 2010). We genetically fused HcT either to linkers 1 or 2 while linker 3 was prepared separately for chemical attachment of a fluorescent cargo. The linkers with or without HcT proteins were expressed in *E. coli* and purified by affinity chromatography and gel-filtration on a Superdex-200 column. Maleimide-Cy3 was used to chemically label the linker 3 via cysteine groups. We then prepared 2xHcT and 1xHcT carrying the Cy3 fluorescent label, named 2xHcT-Cy3 and 1xHcT-Cy3 (Fig. 1a, upper panel, and Fig. 1b). The two molecules were applied to rat cortical neurons and bound fluorescence was imaged and quantified. Fig. 1c shows that 2xHcT-Cy3 exhibited 2.6-fold more internalisation into primary neurons compared to 1xTet-Cy3. We next investigated whether duplication of the tetanus binding domain can enhance neuronal delivery of the botulinum type A protease (LCHn/A). We chose LCHn/A as a cargo because its activity can be conveniently detected by SNAP-25 cleavage with highest possible sensitivity, whereas the cleavage of VAMP protein is more difficult to detect *in situ* (Yadirgi et al., 2017, Gray et al., 2018). After expression in bacteria, LCHn/A fused to linker 3 was purified by affinity chromatography and gel-filtration. Mixing the two HcTs in the presence of LCHn/A-linker3 led to formation of 225 kDa SDS-resistant chimeric protein which we call Bitox/TT (Fig. 1d). Formation of ‘single binding’ Bitox/T was achieved by mixing HcT-linker 1 with LCHn/A-linker 3 in the presence of linker 2 alone, yielding a 175 kDa construct (Fig. 1d). We compared the efficiency of the cleavage of intra-neuronal SNAP-25 by Bitox/TT versus Bitox/T in rat cortical neuronal cultures. Fig. 1e and f shows that Bitox/TT caused 28 fold enhancement of neuronal SNAP-25 cleavage indicating that the duplication of binding domain can lead to enhancement of enzymatic delivery into neurons.

**Fig. 2:**
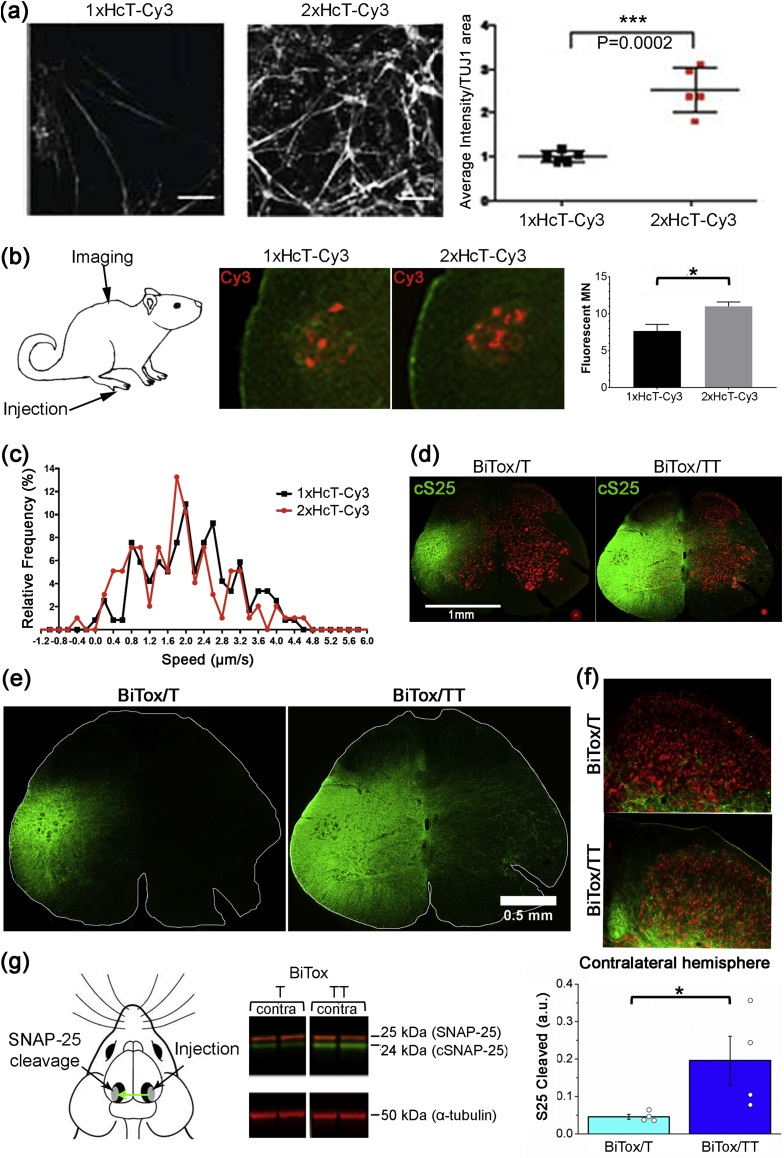
Duplication of tetanus binding domain enhances neuronal delivery of Cy3 and botulinum enzyme in rodents. **a)** Examples of fluorescent micrographs (left) and their quantification (right) of cultured mouse motor neurons treated with either 1xHcT-Cy3 or 2xHcT-Cy3 (10 nM, scale bars, 25 μm). Bar chart shows average intensity normalised to tubulin/TUJ1 signal: 1xHcT-Cy3 = 1 ± 0.1, 2xHcT-Cy3 = 2.5 ± 0.5 (n = 5). **b)** Schematic showing the injection site in mouse hind paw and the spinal cord area where SNAP-25 cleavage was quantified (left panel). Spinal cord micrographs of mice injected intraplantar with either 1xHcT-Cy3 and 2xHcT-Cy3 indicating an increased accumulation of Cy3 label (red) in the ventral horn of the spinal cord in the latter case (middle panel). Bar chart showing 50% increase in the number of fluorescent motor neurons in the most fluorescent section of the spinal cord in the case of 2xHcT-Cy3 (right panel): 1xHcT = 7.67 ± 0.9; 2xHcT = 11 ± 0.6. **c)** Axonal retrograde trafficking of HcT-containing carriers is not affected by duplication of tetanus binding domain. Quantification of retrograde axonal transport was performed in cultured mouse motor neurons pulse-labelled with either 1xHcT-Cy3 or 2xHcT-Cy3 (both 10 nM) in microfluidic devices. Cy3-positive carriers were tracked and their frequency and average speed were plotted in the graph (23 signalling endosomes, 217 transport steps). **d-f)** Example micrographs of whole spinal cords (**d) and (e)** or dorsal horn region (**f)** from rats injected intraplantar with either Bitox/T or Bitox/TT. Tissues were immunostained with cleaved SNAP-25 antibody (green) (**d-f**) and the NeuN neuronal marker (red) (**d) and (f)** (n = 3). **g)** Schematic showing the injection site in the mouse visual cortex and the contralateral site where SNAP-25 cleavage was quantified (left panel). An immunoblot of contralateral visual cortex of mice injected with Bitox/T or Bitox/TT. Note increased immunosignal of cleaved SNAP-25 (green) in the case of Bitox/TT compared to Bitox/T (middle panel). Bar chart showing four-fold increase in the quantified immunosignals of cleaved SNAP-25 in the contralateral visual cortex of mouse brain (right panel) (n = 8).

Next, we investigated the properties of double-binding tetanus molecules in motor neurons, the natural target of tetanus toxin. 2xHcT-Cy3 and 1xHcT-Cy3 were applied to mouse motor neurons and bound fluorescence was imaged and quantified. Fig. 2a shows that 2xHcT-Cy3 exhibited 2.5-fold more binding to motor neurons compared to 1xTet-Cy3, similar to the results obtained in cortical neuronal cultures. Can duplication of HcT cause an enhanced delivery in vivo? To answer this question, 2xHcT-Cy3 (1 μg, 8 pmol) and 1xHcT-Cy3 (1 μg, 13 pmol) were injected into mouse hind paws and after three days spinal cords were sectioned and analysed by fluorescent microscopy. Fig. 2b shows a 50% increase in accumulation of Cy3 signal for the duplicated molecule in the cell bodies of motor neurons in the ipsilateral ventral horn of injected animals despite the lower amount of 2xHcT in the injection. To evaluate if such an increase is due to acceleration of axonal transport, we analysed intraneuronal trafficking in cultured motor neurons (Terenzio et al., 2014). We applied 10 nM 1xHcT-Cy3 and 2xHcT-Cy3 to the chamber containing terminals of primary motor neurons for 30 min and after washing, the axonal retrograde transport of the fluorescent molecules was imaged and quantified in microfluidic devices. The frequency and brightness of the retrograde transport carriers were very similar between the two HcT constructs suggesting that the sorting pathway to these organelles is similar under these conditions (Fig. 2c). It was evident that the dual HcT follows the canonical internalisation pathway and both constructs moved along the microtubules with the same speed (Fig. 2c), suggesting that binding to the neuronal membrane is the critical step. We next investigated whether enhanced uptake of Bitox/TT could result in augmentation of exogenous enzymatic activity in the spinal cord of rats. We measured the botulinum enzymatic activity by quantifying the proteolytic cleavage of SNAP-25 using the neoepitope antibody against the cleaved SNAP-25 (Yadirgi et al., 2017). As observed previously for Bitox/T (Ferrari et al., 2013), injections of Bitox/TT did not induce any noticeable behavioural change with rats moving freely. Analysis of the spinal cord using immunohistochemistry with the neoepitope-SNAP-25 antibody revealed a significant cleavage of SNAP-25 in the ventral horn area surrounding motor neurons in the case of Bitox/T (Fig. 2d). This cleavage was dramatically enhanced in the case of Bitox/TT with almost the whole ipsilateral side of the spinal cord at L4-5 level exhibiting the SNAP-25 cleavage (Fig. 2d-f). Quantification of the percentage of SNAP25-cleaved area relevant to the total section area showed a four-fold increase in the area of penetration of botulinum activity in the case of Bitox/TT: BiTox/T = 15.6 ± 3, BiTox/TT = 44.7 ± 5. Further we investigated retrograde transport after a local injection into the visual cortex of mice. We injected the toxins in the binocular portion of the visual cortex, to maximise callosal neuron uptake (and thus long-range trafficking) (Restani et al., 2009). Three days following injection, we dissected tissues corresponding to injected and contralateral visual cortices (Fig. 2g). Western blots were performed and quantified for the levels of cleaved SNAP-25. Ipsilaterally, at the site of injection, the cleavage of SNAP-25 was strong in both cases (data not shown). However, immunoblotting analysis of the contralateral visual cortex revealed that Bitox/TT caused a four-fold stronger enzymatic action in the contralateral side compared to Bitox/T (Fig. 2g).

We therefore conclude that duplication of tetanus binding domain can efficiently increase delivery of both small molecules and enzymes into neurons both *in vitro* and in vivo.

### 2.2 Duplication of type D botulinum binding domain

**Fig. 3:**
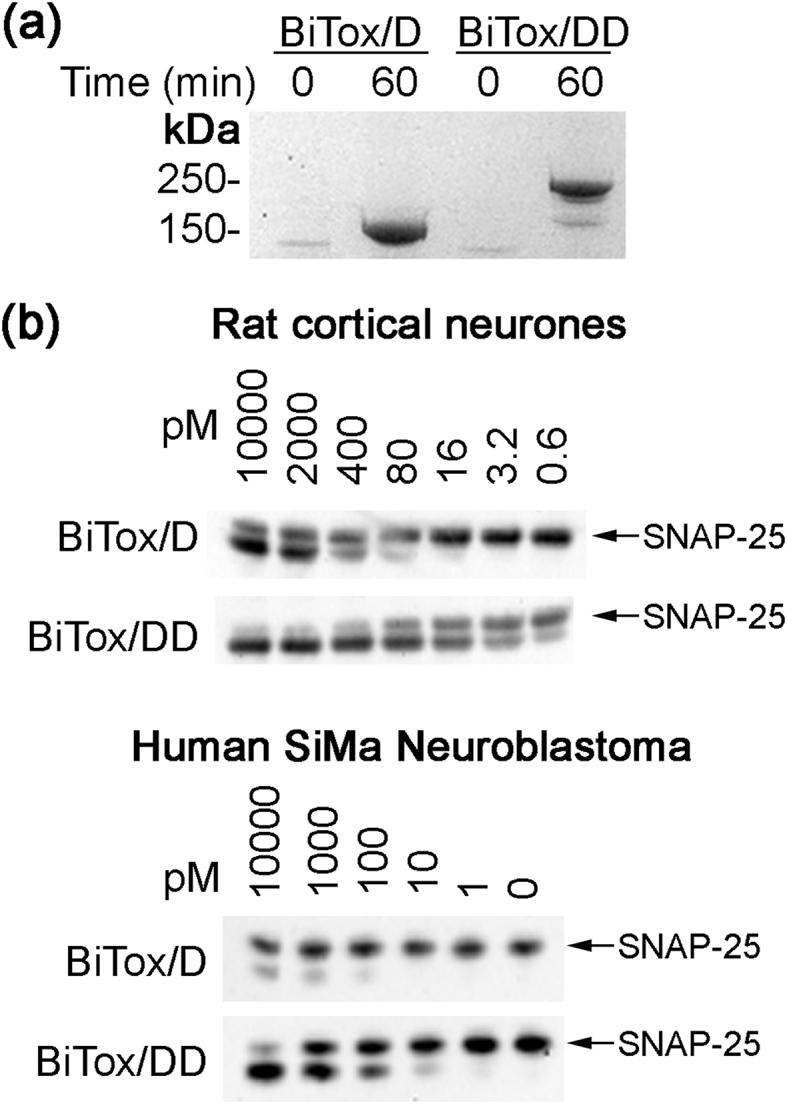
Duplication of botulinum type D binding domain results in augmented delivery of botulinum enzyme into neurons. **a)** Coomassie-stained SDS-PAGE gel showing formation of Bitox/D and Bitox/DD and the difference in their apparent molecular weights. Proteins were analysed in non-reducing conditions and thus exhibit full protein content. **b)** Duplication of HcD within novel botulinum type A stapled chimera increases cleavage of SNAP-25 in both rat cortical neurons (upper panel; BiTox/D EC_50_ = 637 pM, BiTox/DD EC_50_ = 6.4 pM) and human neuroblastoma cells (n = 3).

We next focused on the botulinum protein-based neuronal delivery because humans are not routinely immunised against botulinum neurotoxins in contrast to the global tetanus vaccination programme. Therefore botulinum-based delivery vehicles are more likely to be used in future medicine. BoNTs type A and type B are well known therapeutics already used to cause temporary muscle paralysis or reduce excessive secretory functions (Ovsepian et al., 2019). To avoid potential immune cross-reaction with type A and type B botulinum drugs, we decided to focus on other types of botulinum binding domains. We chose type D botulinum binding domain in order to devise an enhanced neuronal delivery system as our previous study demonstrated the potential of BoNT/D as a neuronal delivery vehicle (Bade et al., 2004). We expressed Hc type D, HcD, fused to linkers 1 and 2 similar to the tetanus HcT domain. For evaluation of neuronal delivery of enzymatic activity, we exploited LCHn/A fused to linker 3. Upon assembly, the SDS-PAGE gel reveals a BiTox/DD having molecular weight of ~225 kDa compared to 175 kDa BiTox/D which can be accounted by the presence of an additional HcD in the former (Fig. 3a). The activity of BiTox/DD was evaluated in rat cortical neuronal cultures and differentiated human SiMa neuroblastoma cells which were shown previously to bind BoNTs type A to C. Direct comparison of SNAP-25 cleavage by BiTox/D and BiTox/DD revealed a strong enhancement of SNAP-25 cleavage in both human and rat neurons (one hundred fold) (Fig. 3b).

### 2.3 BoNT/C with enhanced binding funding

**Fig. 4:**
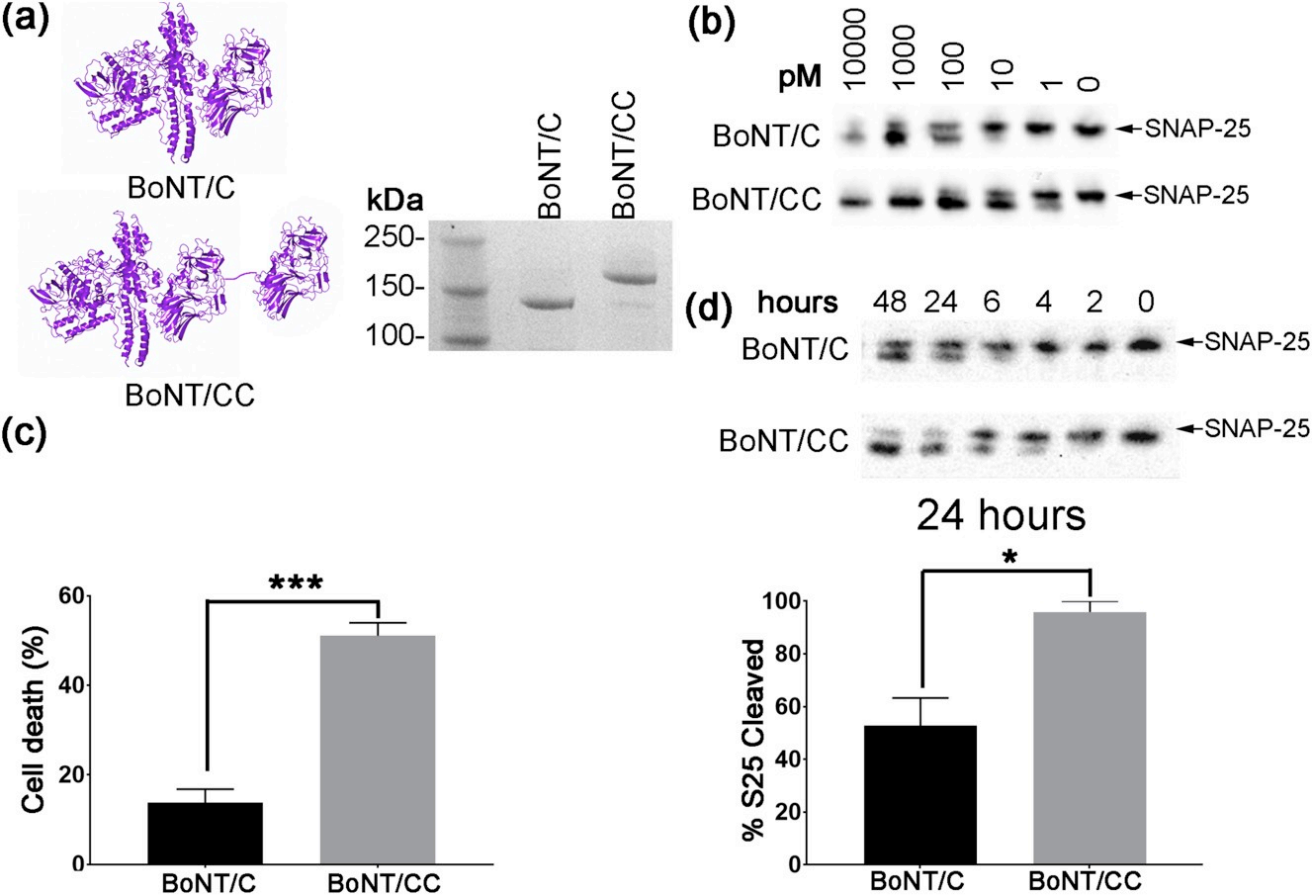
Recombinant duplication of the receptor-binding domain of BoNT/C (BoNT/CC) results in an enhancement of botulinum functions. **a)** Schematic (left panel) and Coomassie-stained SDS-PAGE gel (right panel) showing the difference between BoNT/C and the novel BoNT/CC. Proteins were analysed in non-reducing conditions and thus exhibit full protein content. **b)** Immunoblot of SiMa neuroblastoma cells treated with either BoNT/C or BoNT/CC at indicated concentrations reveals a five-fold enhancement of SNAP-25 cleavage by the duplicated molecule (BoNT/C EC_50_ = 106 pM, BoNT/CC EC_50_ = 20.4 pM) (n = 3). **c)** Quantification of cell death in differentiated SiMa neuroblastoma cell culture treated with 2 nM of BoNT/C or BoNT/CC indicates three-fold increase in cytotoxic properties of the latter (BoNT/C = 13.8%, BoNT/CC = 53.2%) (n = 3). **d)** Immunoblot of SiMa neuroblastoma cells treated with 3 nM either BoNT/C or BoNT/CC for indicated duration of time, with quantification of cleaved SNAP-25 at 24 h shown in the bar chart (lower panel) (n = 3).

The enhanced neuronal action upon duplication of tetanus and botulinum D binding domains raises the question on whether it is possible to improve native full botulinum neurotoxins for medical use. Among the botulinum family of neurotoxins, BoNT/C and BoNT/D do not cause botulism in humans by ingestion (Rossetto et al., 2014) thus offering a degree of safety when engineering novel botulinum configurations for exploring neuronal delivery of macromolecules. We attached a second HcC linked via a forty amino acid-flexible linker to the native BoNT/C yielding a single recombinant fusion protein (Fig. 4a). As an additional security step, we introduced a thrombin-activation site into the BoNT/C structure, just after the light chain, such that the molecule is fully active only after addition of thrombin as previously reported (Darios et al., 2010). Fig. 4a shows the migration behaviour in an SDS-PAGE gel of the native BoNT/C and BoNT/CC, the latter carrying duplicated HcC and hence exhibiting a larger molecular weight. Direct comparison of the efficiency of SNAP-25 cleavage in SiMa neuroblastoma cells by the native BoNT/C and engineered BoNT/CC revealed a significant enhancement of SNAP-25 cleavage in the latter case, estimated to be 5 fold (Fig. 4b). We and others reported that BoNT/C causes apoptosis of cultured neurons most likely due to elimination of plasma membrane SNAREs, which likely affects vesicle traffic required for nutrient uptake (Rust et al., 2016). To investigate a functional consequence of duplication of HcC we analysed neuronal survival in the presence of BoNT/C and BoNT/CC. After 65 h incubation, the Deep Blue cell viability assay reagent was added to the neuroblastoma cell media, and the fluorescent signal was acquired at required wavelengths, Ex 560 nm/Em 590 nM. Fig. 4c shows that BoNT/CC caused a three-fold increase in cell death compared to native BoNT/C (BoNT/CC – 53.2%; BoNT/C – 13.8%). This result provides additional evidence that duplication of botulinum binding domains affords enhancement of botulinum activities within neurons. Finally, we investigated whether the kinetics of cleavage of SNAP-25 in SiMa neuronal cultures is accelerated upon duplication of HcC. Fig. 4d shows BoNT/CC caused cleavage of the majority of intracellular SNAP-25 after 24 h whereas native BoNT/C at similar concentration of 3 nM cleaved only half of SNAP-25. Quantification of SNAP-25 cleavage in three separate experiments revealed that duplication of the binding domain led to a two-fold acceleration of SNAP-25 cleavage in SiMa neuronal cultures.

## 3 Discussion

Novel ways to deliver imaging reagents and therapeutics to neurons will provide new opportunities to study the neuronal function in health and disease and will also allow better treatment of neurological diseases. More effective and better-tolerated pharmacological therapies for neurological disorders are urgently needed in the era of rapidly ageing population. Neurons are unique in that they are non-dividing cells with limited ability to renew their functions in disease or during the ageing process. Current treatments for neurological disorders often rely on systemic administration, resulting in multiple adverse effects due to off-target drug distribution, as exemplified by opioid pain-killers (Stein, 2018). Another major obstacle in neurological treatment is the blood brain barrier (BBB), which prevents large molecules from entering the CNS from the vascular system (Ovsepian et al., 2016). Because of the BBB, achieving physiologically relevant doses of drugs in neural tissue by systemic delivery may require high, sometimes toxic, doses. To overcome these difficulties new delivery methods which circumvent the BBB and deliver drugs via peripheral neural pathways are under investigation.

Here we explored, for the first time, the effects of duplication of neural-binding domains derived from the clostridial neurotoxins on neurons *in vitro* and in vivo. Tetanus and botulinum neurotoxins are known to be the most potent agents to bind neurons causing spastic and flaccid paralysis, respectively, at nanogram doses (Surana et al., 2018). They act by cleaving the SNARE proteins, thereby causing months-long nerve block (Rossetto et al., 2014). Without their SNARE-proteolytic action, these neurotoxins can be converted into powerful neural delivery vehicles. This can be achieved by inactivating the proteolytic clostridial enzymes via point-mutations as was demonstrated recently for BoNT/A and BoNT/C (Vazquez-Cintron et al., 2014, Vazquez-Cintron et al., 2017). Importantly, in the case of the inactivated full-length neurotoxin the presence of the translocation domain permits drug delivery, via vesicular escape route, into the neuronal cytosol (Bade et al., 2004, Vazquez-Cintron et al., 2017). Alternatively, the structurally-independent neuronal binding domains of clostridial neurotoxins (Hc domain alone) can be produced separately and utilised for therapeutic or imaging cargo delivery into neurons (Surana et al., 2018). In the latter case, the Hc domain with the attached cargo would be retained in the endolysomal compartments and drugs/imaging agents will be released into the cytosol after proteolytic degradation.

The tetanus neuronal binding domain has been utilised by many group to demonstrate successful delivery of imaging agents and therapeutics into the CNS after peripheral injection (Toivonen et al., 2010, Bergen et al., 2008). One problem for translation of tetanus-based drug delivery approach lies in the fact that humans are immunised against tetanus (Yen and Thwaites, 2019). This necessitates the use of smallest amounts possible to avoid triggering an immune response. However, it is possible that even the smaller amounts of tetanus Hc-based delivery agents are still non-functional in immunised humans. Even so, the utility of our current observation may still be beneficial for the hundreds of millions of non-immunised humans and possibly in veterinary medicine.

We demonstrated here that duplication of the tetanus binding domain allows increased efficiency of delivery of an imaging reagent and a model enzyme, LC type A, into the spinal cord. This is likely not due to an acceleration of the transport of this chimeric protein but rather to the increase in binding to the neuronal membrane, since we have not observed any increase in accelerated intraneuronal trafficking. It is more likely that there is an increase in internalisation rates of duplicated clostridial molecules from the plasma membrane since many receptor mechanisms exhibit enhancement in endocytosis in the case of dual binding (Vauquelin and Charlton, 2013). It will be important to address the enhancement mechanisms in future studies using advanced binding and imaging methods with high rate kinetic capabilities. Previously, it was proposed that the light chains and translocation domains of clostridial neurotoxins, acting in conjunction with binding domains, dictate their local or distant destinations (Wang et al., 2012). Indeed, our study revealed a massive cleavage of SNAP-25 induced by Bitox/TT in the spinal cord neurons beyond the ventral horn but surprisingly with no visible effects on the motor behaviour of rats. Previously, a similar molecule called Tetbot was tested in rats at similar doses without any impediment of motor functions (Ferrari et al., 2013). It will be important to identify in future studies the nature of neurons which are targeted by the new chimeras and perform extensive studies of motor function, as it may be relevant to treatment of particular neurological disorders.

Our data also show that duplication of botulinum binding domain derived from BoNT/D allows a high magnification of botulinum enzymatic activity delivered into human- and rat-derived neurons, raising the possibility that future botulinum therapeutics could be improved by simply adding an additional binding domain. We tested this possibility by remodelling the native BoNT/C with addition of the second HcC domain by direct fusion, rather than our trimeric assembly system. Our *in vitro* experiments revealed enhanced SNAP-25 cleaving ability of BoNT/CC versus the native toxin. We have not conducted in vivo studies with recombinant BoNT/CC due to regulatory restrictions but we envisage future translational studies in collaboration with industry. This is medically relevant since BoNT/C has been proposed as an alternative to the long-acting BoNT/A. Another benefit of duplication of botulinum binding domains is that the cleavage of SNAP-25 was accelerated in neuronal cultures which might translate into a therapeutic advantage but this possible acceleration needs to be also investigated in future physiological studies.

Overall, we demonstrated that the duplication of the clostridial neuron-binding domains leads to increased delivery of enzymatic and imaging cargoes into neurons. With regards to future explorations, duplication of binding domains could be of homologous or heterologous nature, where binding domains can be coupled from different botulinum serotypes. It might be also possible to further increase intraneuronal delivery by adding more than two neuronal binding domains. This interesting possibility will need to be explored. Such enhanced botulinum therapeutics should achieve therapeutic effects with lower doses compared to the current doses of these bacterially-derived immunogenic molecules, especially where high doses are required, for example in the cases of severe migraine and cerebral palsy. Pertinently, a family of patents by Allergan highlighted possible therapeutic applications of multivalent botulinum neurotoxins (Steward et al., 2007). Finally, the enhanced botulinum therapeutics shall exhibit faster therapeutic effects benefitting many neurological patients under treatment with conventional botulinum drugs.

## 4 Methods

### 4.1 Ethics statement

All experimental procedures conformed to the European Communities Council Directive number 86/609/EEC. We have followed the rules of Three R's to reduce the impact of research on animals. Animals were reared in a 12 h light/dark cycle, with food and water available ad libitum. Animal work was carried out under project licences in accordance with the Animals (Scientific Procedures) Act 1986 and its associated guidelines.

### 4.2 Protein production

With the exception of BoNT/C-based proteins, all recombinant proteins were made as glutathione-S-transferase (GST) C-terminal fusions cleavable by thrombin in the pGEX-KG expression vector and expressed in *E. coli* BL21-Gold-PLysS-DE3 (Agilent). Expressed proteins were purified by glutathione affinity chromatography and eluted from the glutathione beads using thrombin as described previously (Darios et al., 2010). The linker 1 sequence is based on the sequence of rat VAMP2 (2–84, UniProt P63045). Fusions of the linker 1 with the tetanus binding domain (HcT- 856–1315, Uniprot P04958) and the Botulinum binding domains type D (HcD- 865–1275, Uniprot P19321) were made by inserting the DNA sequence for VAMP2 (2–84, Uniprot P63045) into the XhoI site of the pGEX-KG vector and the DNA sequence for the relevant binding domain into the SacI site. The linker 2 was based on the SNARE helix of rat syntaxin 3 (195–253, Uniprot Q08849). Linker 2- HcT and linker 2-HcD, were each designed by inserting the DNA sequence for rat syntaxin 3 (195–253, Uniprot Q08849) into the XbaI site of the pGEX-KG vector and the DNA sequence of the relevant binding domain into the SacI site. The enzymatic portion of the botulinum type A1 neurotoxin consisting of its light chain and translocation domain fused to linker 3 (based on the sequence of SNAP-25, aa 1–206) was prepared as previously described (Darios et al., 2010). For preparation of Cy3 molecules, the sequence for SNAP-25 was inserted into the BamHI site of the pGEX-KG plasmid. After protein expression, Cy3-NHS was conjugated to the free cysteines of SNAP-25 at 8-fold molar excess. Conjugated linker 3-Cy3 was then purified by gel filtration on Superdex-200 column. The single-Hc constructs were assembled using a synthetic syntaxin 3 peptide as in (Darios et al., 2010).

### 4.3 Formation of single- and double-ligand clostridial constructs

Fusion proteins containing an eqimolar ratio of linker 1, linker 2 and linker 3 components were mixed in Buffer A (20 mM HEPES, 100 mM NaCl_2_ and 0.4% octyl glucoside) and were left at 20 °C for 1 h to allow formation of the SNARE helical bundle. Irreversible assembly of protein complexes was confirmed by sodium dodecyl sulphate–polyacrylamide gel electrophoresis (SDS–PAGE). The gels were run at 4 °C and proteins were visualised by Coomassie Blue staining, as described previously (Darios et al., 2010).

### 4.4 Expression of BoNT/C and BoNT/CC

pBoNT/Cs-thro was generated by inserting the full-length open reading frame for BoNT/C (Uniprot P18640) as an EcoRI-PstI fragment into pQE3 (Qiagen). It encodes in addition a Strep-tag, PGWSHPQFEK, following the C-terminal codon and an *E. coli* protease and thrombin sensitive peptide, SKTKSLVPRGS, replacing D442-N448 between LC and the heavy chain. pBoNT/CC was generated based on pBoNT/Cs by inserting a DNA segment encoding the flexible 40 aa linker, PGASGGGGASSAGGGSSAGSGSSGGGAAAGSGASGSASGS, between the C-terminal end, followed by a copy of the Hc-fragment encoding part of BoNT/C, (867–1291), and the Strep-tag coding segment. Recombinant full-length BoNT/C proteins were purified from the *E. coli* strain M15pREP4 (Qiagen GmbH, Hilden, Germany), following 16 h of induction at 21 °C. Proteins were purified on Streptactin-Superflow beads (IBA GmbH), according to the manufacturer's instructions and eluted in 100 mM Tris–HCl, pH 8.0, 10 mM desthiobiotin. Both protein preparations were treated with thrombin-attached beads in the presence of 10 mM CaCl_2_ for 2 h at 21 °C. Protein was collected in the flow-through and stored at −80 °C.

### 4.5 Cortical neuron cultures

All cells and cell lines were maintained at 37 °C and 5% CO2. Cortical neurons were dissected from embryonic day 17.5 rat pups and washed in Hanks' Balanced Salt Solution (HBSS), before treating with 70 μL of 2% Trypsin (Sigma) for 15 min at 37 °C, followed by addition in 5 μg of DNAse (Sigma). Cells were resuspended in 1 mL triturating solution (1% albumax (Gibco), 0.5 mg/mL Trypsin inhibitor (Sigma), 1 μg/mL DNAse in HBSS). Cells were triturated with 3 progressively smaller glass pipettes before addition of cortical media up to 5 ml. 50,000 cells in 150 μL media were plated on 96 well plates coated with poly-D-lysine. Cells were maintained in Neurobasal medium (Gibco) supplemented with 1% B27 (Gibco), 1% Penicillin/streptomycin (Gibco), and 400 μM L-glutamine (Gibco). Half of the medium was changed every 3–4 days and cultures were tested at least 1 week after plating and kept for a maximum of 4 weeks.

### 4.6 Neuroblastoma cell cultures

Media for the SiMa cell line contained 90% DMEM and 10% FBS. SiMa neuroblastoma cell differentiation medium contained 96% RPMI, 10 mM HEPES pH 7.8, 1X non-essential amino acids (Gibco), 2% B27 (Gibco) and 10 μM all trans-retinoic acid (Sigma). SiMa cells were differentiated by plating onto laminin-coated wells in a 96-well plate, at a density of 20,000 cells per well, in 150 μL of differentiation media (Rust et al., 2016). SiMa cells were used in experiments 72 h after induction of differentiation.

### 4.7 Motor neuron cultures

Mouse spinal cord motor neurons were isolated from E13-14 embryos and plated on poly-D-ornithine and laminin-coated 24 well plates, glass coverslips or 35-mm glass-bottom microwell dishes (MaTeK corporation). Motor neurons were cultured for between five and eight days in media containing Neurobasal medium supplemented with 2% B27, 2% heat-inactivated horse serum, 1% GLUTAMAX, 25 μM 2-mercaptoethanol, 10 ng/mL rat ciliary neurotrophic factor (CNTF; R&D Systems), 100 pg/mL rat glial cell line-derived neurotrophic factor (GDNF; R&D Systems), 1 ng/mL brain derived neurotrophic factor (BDNF; R&D Systems) and 1% penicillin/streptomycin.

### 4.8 Neuronal labelling

Primary motor neurons were incubated with 10 nM 1xHcT-Cy3 or 2xHcT-Cy3 for 30 min. Motor neurons on coverslips were fixed in 4% PFA in phosphate buffer saline (PBS) for 15 min at 22 °C. Coverslips were then washed with PBS and permeabilised and blocked for 10 min in a solution of 5% BSA and 0.1% Triton X-100 in PBS. Primary antibodies were diluted in 5% bovine serum albumin (BSA) in PBS and incubated for 1 h at 22 °C. Coverslips were washed 3 times in PBS, then incubated with the appropriate fluorescently conjugated secondary antibodies diluted in 5% BSA for 1 h at 22 °C. Finally, coverslips were washed 3 times with PBS, once with water and then mounted using Mowiol-488. Coverslips were imaged with an invert Zeiss LSM 780 confocal microscope using a 63X Plan-Apochromat oil immersion objective with an NA of 1.4. Immunofluorescence staining was quantified using ImageJ. For live imaging, primary cortical or motor neurons were incubated with 10 nM 1xHcT-Cy3 or 2xHcT-Cy3 for 30 min. Cultures were washed and fresh media was applied before imaging on an epifluorescence microscope.

### 4.9 Measurement of axonal retrograde transport

Primary motor neurons were incubated with 10 nM 1xHcT-Cy3 or 2xHcT-Cy3 for 30 min. Cultures were washed and fresh motor neuron media was applied. Cells were then imaged using a Zeiss LSM 780 microscope equipped with a Zeiss X63, 1.40 NA DIC PlanApochromat oil-immersion objective whilst kept at 37 °C. Retrograde transport was quantified using Motion Analysis software (Kinetic Imaging) as done previously (Gibbs et al., 2018).

### 4.10 Investigation of Cy3-labelled fluorescent constructs in mouse spinal cord

2 months old Bl6/CJ male mice were anaesthetized using isoflurane followed by intraplantar injections of fluorescent HcT constructs into the right footpad (1 μg equivalent to 13 pmol for 1xHcT and 8 pmol 2xHcT) in a volume of 5 μl saline. Left foot served as negative control. 48 h after injection mice were perfused with 4% PFA, spinal cords isolated by laminectomy, post-fixed for 4 h in the fixative. Then samples were rinsed in PBS, and vibrotome slices of the L4-S2 spinal cord slices were cut to 60 μm thickness. On average, 8 sections from each spinal cord sample had some degree of fluorescent signal in the right hemisphere. Sections with the highest fluorescent signal were selected from each mouse to count total number of fluorescently labelled motor neuron cell bodies per section.

### 4.11 Investigation of BiTox/T and BiTox/TT in mouse visual cortex

2 months old mice were injected with 3 ng of Bitox/TT (13.3 fmol) or Bitox/T (17.1 fmol) into left visual cortex (coordinates: 3 mm lateral and in correspondence with lambda). Right and left visual cortices were dissected three days following injections. Proteins were extracted with lysis buffer (1% Triton X-100, 10% glycerol, 20 mM Tris-HCl, pH 7.5, 150 mM NaCl, 10 mM EDTA, 0.1 mM Na_3_VO_4_, 1 μg/mL leupeptin, 1 μg/mL aprotinin, and 1 mM PMSF), and the total concentration of the samples was assessed with a protein assay kit (Bio-Rad) using BSA as a standard. Immunoblotting was performed as described previously (Spalletti et al., 2017). Protein extracts were separated by electrophoresis and then immunoblotted. Membranes were incubated with 1:5000 dilution of either anti-cleaved SNAP-25 or anti-intact SNAP-25 primary antibody overnight at 4 °C. Anti-cleaved SNAP-25 was raised against a synthesized peptide of SNAP-25 190–197 (TRIDEANQ). For detection of native SNAP-25 we used rabbit polyclonal antibody against the native uncleaved SNAP-25 protein. Membranes were also probed with anti-alpha tubulin antibody (mouse monoclonal, 1:15,000 dilution; Sigma) as an internal standard for protein quantification. Blots were then rinsed in PBS with 0.2% Tween 20, incubated in infrared labelled secondary antibodies (anti-mouse IRDye 680LT at 1:30,000 or anti-rabbit 800CW at 1:20,000; Li-Cor Biosciences), and rinsed in PBS. Membranes were scanned using an Odyssey IR scanner (Li-Cor Biosciences), and densitometry analysis was performed with Image Studio software version 3.1 (Li-Cor Biosciences). The band of interest was determined with respect to protein molecular weight standards. Antibody signal was calculated as integrated intensity of the region defined around the band of interest. Protein amount of cleaved SNAP-25 and intact SNAP-25 was normalised relative to the alpha-tubulin immunosignal.

### 4.12 Investigation of BiTox/T and BiTox/TT in rat spinal cord

300 ng of single- (1.7 pmol) and double-liganded (1.3 pmol) botulinum constructs were injected into the left hind paw of rats. Group 1 (3 rats) received BiTox/T whilst Group 2 (3 rats) received BiTox/TT. 6 days post injection, rats were sacrificed and perfused with 4% PFA. The spinal cords were removed and sectioned for immunohistochemistry. Spinal cord sections were incubated with anti-cleaved SNAP-25 antibody (1:10000 diluted in PBS) and left overnight on a rocker at 22 °C. The following day, the sections underwent three PBS washes before incubation with biotinylated secondary antibodies (1:400 goat anti-rabbit, Vector Stain) for 90 min at 22 °C as part of a Tyramide Signal Amplification (TSA) protocol. After additional three washes, sections were incubated in ABC complex (1:125; Vector Stain, ABC elite kit, Vector Labs) for 30 min at 22 °C. After three washes with PBS, sections were incubated with biotinylated tyramide (1:75; TSA Stain Kit; PerkinElmer) for 7 min. The tissue sections were imaged using a Leica DM IRB epifluorescence microscope with MicroManager software. Image analysis and quantification was performed using FIJI software for particle analysis.

### 4.13 Western blotting analysis of botulinum activity in neuronal cell cultures

Molecules containing botulinum type A or C protease were compared in differentiated neuroblastoma or cortical neurones for the cleavage of intraneuronal SNAP-25, which can be detected as a shift in the molecular weight of SNAP-25 in SDS-PAGE gels. Immunoblotting was done using an in-house rabbit polyclonal SNAP-25 antibody which recognises both the intact and cleaved SNAP-25. Following treatment of cortical neurons or differentiated SiMa neuroblastoma cells with botulinum constructs for 65 h (unless otherwise stated) in 96 well plates, cell culture media was removed before addition of SDS-PAGE loading buffer (56 mM sodium dodecyl sulphate, 0.05 M Tris-HCl, pH 6.8, 1.6 mM EDTA, 6.25% glycerol, 0.0001% bromophenol blue, 10 mM MgCl_2_, 26 U/mL benzonase). Plates were then shaken at 900 rpm for 10 min. Samples were boiled for 3 min at 95 °C and then run on 12% Novex SDS–PAGE gels (Invitrogen). For assessment of intraneuronal SNAP-25 cleavage, gel running time was 2 h to increase separation between cleaved and intact SNAP-25. Following separation, proteins were transferred onto Immobilon-P membranes, and then incubated for 30 min in blocking solution (5% milk, 0.1% Tween 20 in PBS). The rabbit SNAP-25 polyclonal antibody was added at 1:3000 dilution to the blocking solution at 4 °C overnight. Membranes were washed three times in 0.1% Tween 20 in PBS for 5 min and then incubated for 30 min in the blocking solution containing secondary peroxidase-conjugated donkey anti-rabbit antibodies (Amersham). Membranes were washed three times for 5 min in 0.1% Tween 20 in PBS. Immunoreactive protein bands were visualised using SuperSignal West Dura solution (Thermo Scientific) on a BioRad imaging station and by exposure to X-Ray films (Fuji, UK). Protein bands were quantified using the Quantity One software (BioRad). EC_50_ values were calculated using the non-linear fit equation in GraphPad Prism.

### 4.14 Neuroblastoma cytotoxicity assay

The cytotoxic effect of molecules containing the type C protease were compared in differentiated neuroblastoma using the Deep Blue Cell Viability Kit (BioLegend). Following treatment of differentiated Nanoluc-VAMP2 expressing SiMa neuroblastoma with botulinum constructs for 65 h in 96 well plates, 15 μL of Deep Blue Cell Viability reagent was added to each well before incubation at 37 °C for 6 h. Fluorescence of the metabolised reagent was measured at Excitation 560 nm and Emission 590 nm. Wells containing differentiation media alone were used as a negative control. Cell viability was normalised using the mean average of untreated cells as the 100% value.

## Statistical analysis

All experiments were repeated at least three times and significance was determined using unpaired *t*-test.

## Ethics statement

The authors declare that all experimental procedures within this paper conformed to the European Communities Council Directive number 86/609/EEC. They have followed the rules of Three R's to reduce the impact of research on animals. Animal work was carried out under project licences in accordance with the Animals (Scientific Procedures) Act 1986 and its associated guidelines.

## Author contributions section

Charlotte Leese: Methodology, Validation, Investigation, Resources, Writing - Original Draft, Visualization. Rebecca Bresnahan: Investigation. Ciara Doran: Investigation, Visualization. Deniz Simsek: Investigation. Alexander Fellows: Investigation. Laura Restani: Investigation. Matteo Caleo: Methodology. Giampietro Schiavo: Methodology, Investigation. Timur Mavlyutov: Methodology, Investigation. Tina Henke: Investigation. Thomas Binz: Methodology, Investigation, Resources. Bazbek Davletov: Conceptualization, Methodology, Writing - Original Draft, Writing - Review & Editing, Supervision, Project administration, Funding acquisition

## Declaration of competing interest

The authors have no competing interests to declare.
